# Assessment of food consumption, smoking, alcohol use, and physical activity by sex and major of study among a sample of college students in Jordan

**DOI:** 10.1016/j.heliyon.2023.e16405

**Published:** 2023-05-24

**Authors:** Nahla Mansour Al Ali, Fatima Khazaal Khazaaleh

**Affiliations:** a Associate Professor Community and Mental Health Department/Faculty of Nursing Jordan University of Science & Technology P.O.Box:3030 Irbid 22110 Jordan; b Community and Mental Health Department/Faculty of Nursing, Jordan University of Science & Technology, P.O.Box:3030 Irbid 22110 Jordan

**Keywords:** Health risk behaviors, Physical activity, Sex, Toxic substance, Food consumption, Smoking, Waterpipe

## Abstract

This study aimed to assess the food frequency and health-related risk behaviors based on gender and major of study (health and non-health majors) in a sample of undergraduate university students. A cross-sectional study was conducted among 708 undergraduate university students (37.4% males; 62.6% females) with a mean age of 20.88(SD = 2.20), ranging from 18 to 37 years from five universities. A self-reported questionnaire was administered in the university classrooms to collect data. Results showed that 47.3% of students reported consuming fruits and vegetables (38.1%), and 54% reported consuming unhealthy foods at least once in the preceding 24 h. About 16% of the students were cigarette smokers, 17% were waterpipe smokers, and slightly more than 4% were alcohol consumers. Significant associations were found between the participants based on their sexes, and study majors (P < 0.001). Male students smoked and consumed alcohol at significantly higher rates than female students. As with regards to the physical activity in the preceding week, students had not carried out any stretching activities (52.8%), strengthening activities (62.4%), cycling (35%), or taken part in any physical activity classes (68.4%). The rates of physical activity were significantly higher among male students than female students (*p* < 0.001). The results showed that non-health major students differ from those in health majors in the consumption of cigarettes (*p* < 0.001) and waterpipe smoking (*p* = 0.027). Students in non-health majors were more likely than students in health majors to have carried out stretching activities (*p* = 0.021) and participated in physical activity classes (*p* = 0.02). Our findings highlighted the importance of identifying health-related risk behaviors among university students to develop a health-promoting intervention tailored to a specific group of students considering their sex and study majors.

## 1 Introduction

Health-risk behaviors such as smoking, alcohol use, physical inactivity, and low fruit and vegetable consumption are prevalent in adolescence and early adulthood, constituting most university student population [1]. Health-risk behaviors are activities carried out by persons with a recurrence or intensity that increase the risk of injury or disease [2].

University students are challenged to make healthy food choices, and their lifestyles are a key indicator of their health in adulthood [3,4]. University life is a transitional stage for many students, providing their first experience of feeling independent without family supervision [5,6]. The university experience can provide students with the opportunity to gain knowledge and perspectives, develop identities, and achieve personal development. Still, it can also lead to intense changes in their lives due to academic, emotional, social, psychological, or financial difficulties [7,8].

Risk behaviors are the most severe risk factors for adolescents’ and young people’s physical and mental health [9]. A study among university students in Jordan found that depressive symptoms were positively correlated with tobacco use, painkillers, inhalants, and heroin use [10]. Risk behaviors are associated with the risk of chronic disease, premature mortality, and disability and negatively impact the physical and mental health of individuals [[11], [12], [13]]. Tobacco use, alcohol consumption, physical inactivity, and insufficient intake of fruits and vegetables are among risky behaviors [11,14,15]. Alcohol consumption and drug use among university students account for 2 and 7% of disability-adjusted life years (DALY), respectively, among individuals from 15 to 24 years of age [16]. Globally, more than 80% of adolescents and 1 in 4 adults were physically inactive [17].

The prevalence of risky behaviors among university students in Saudi Arabia was 47.35%, with 28% being smokers and 66.4% being physically inactive. The age between 18 and 20 years was associated with higher drifting rates, fast driving, and physical inactivity [18]. A systematic review of 38 studies found that the highest prevalence of waterpipe and tobacco smoking was among youth across countries, specifically the Arab population. The prevalence of current waterpipe smoking among university students was highest in the Arabic Gulf region (6%), followed by the United Kingdom (8%), the United States (10%), Syria (15%), Lebanon (28%), and Pakistan (33%). Waterpipe smoking was also high in Lebanon (5%) and Egypt (11%–15%) [19].

Several factors can negatively predict students’ risk behaviors [[20], [21], [22], [23]]. Findings indicated that gender is linked to health risk behaviors engagement, with female students more likely to engage in healthy dietary behaviors and have a low rate of alcohol consumption [24]. In contrast, males engage in physical activity more than females [25]. A study among university students in Jordan showed that male and female students had different use of alcohol and smoking tobacco, with only 5.6% of females drinking alcohol and 94.4% of males smoking tobacco [26]. Also, students’ major was associated with risk behaviors, but nursing students had healthier behaviors than other faculties [27]. Health faculty students engage in healthy behaviors more than humanities and scientific faculty students [28], while religion and law faculties have a low prevalence of all risk behaviors [20].

Emerging adulthood is a time of dramatic life changes and meaningful life choices, which can lead to increased health-risk behaviors [29]. Universities should promote health and well-being by supporting active learning and education, which predicts lifelong health and quality of life [30,31]. In Jordan, undergraduate and postgraduate students are expected to increase by nearly 100,000 in 2030 [32,33], and 90% have a positive view of their health. Smoking is the most negative factor affecting youth’s health, with 25% of male adolescents smoking on a daily basis [34]. A study among 750 students showed that most had eaten less than the recommended daily intake of fruits, vegetables, and milk, while the intake of soft drinks was higher than recommended. One-fifth of students had been physically active for at least 60 min daily, 55.5% had tried smoking, and 44.0% had smoked water pipe [35].

The 10.13039/100004420United Nations Population Fund (UNFPA) Jordan started its Emergency Program in 2012 to promote healthy lifestyles and respond to the needs of young people [36]. Many programs are already running in schools under the name of healthy schools; however, university students are often neglected in health promotion programs, despite the benefits of healthy behaviors [28]. Therefore, determining the risky behaviors of university students and the associated factors provides valuable information for designing appropriate intervention programs for promoting students’ health. This study aimed to assess the pattern of health risk behaviors among undergraduate students in Jordan and determine whether group difference based on sex and study majors exists in these behaviors.

## 2 Methods

### 2.1 Sample and sampling procedure

A cross-sectional design was employed to recruit undergraduate students from all five universities in Irbid City and Al-Mafraq in Jordan (three public universities and two private universities). Undergraduate students (males and females) enrolled full-time in any of the selected universities from different majors and levels of study were approached and invited to participate in this study.

The total number of undergraduate students in the selected universities from 2015 to 2016 was 78069 (43630 females, 34439 males). A sample size calculation was undertaken using the Website http://www.raosoft.com/samplesize.html. A 5% margin of error was selected, along with a 95% confidence interval and 50% response distribution. The calculated sample size was 383 students; about 766 students were approached from five universities. The number of students was chosen from each university according to the distribution rate of students in the universities.

### 2.2 Data collection procedure

Ethical approval was obtained from the Committee on Human Subjects Research at the Jordan University of Science and Technology (No. 33/96/2016, dated May 24, 2016). Before data collection, permission from the selected universities was also obtained. Data was collected using an anonymous self-administered questionnaire in the university classrooms during the 2016/2017 academic year. Classes with many students registered in elective courses were randomly selected using a simple random sampling method. The professors of the courses were contacted to set an agreeable time to administer the survey. Students were approached after class and asked to participate in the study. Students who agreed to participate were asked to sign an informed consent form explaining the study’s nature, purpose, potential risks, and procedures. Completed questionnaires were put in an envelope and were collected by the principal investigators (PIs) in the classroom.

### 2.3 Instruments

The Arabic version of the self-report questionnaire developed by Salameh et al. [37]. was used to collect data from the students. This instrument comprises four different sections.

#### 2.3.1 Sociodemographic information

This section consists of five items on gender, major of study (healthcare-related or non-healthcare-related), marital status, level of study, and type of university (i.e., public or private).

#### 2.3.2 Food consumption in the last 24 h

This section contains seven items asking respondents to report the frequency with which they consumed seven food items (fruit, fruit juice, green salad, cooked vegetables, burger and sausage, French fries or potato chips, and cookies or cake) the previous 24 h. Items are measured on a three-point scale (i.e., No, Yes, once, and Yes, twice or more).

#### 2.3.3 PhysicalActivity and sedentary behaviors

This section includes fourteen items that assess sedentary and physical activity. The first and second questions asked students whether they participate in sports (yes/no) and how many times per week. The second section (which included eight activities such as watching television, computer use, studying/school or work, quiet activities, walking, and riding public transportation) asked students to indicate how many hours per day they did any of the listed activities. These activities were graded on a three-point scale: 1 (less than 1 h), 2 (1–2 h), and 3 (more than 2 h). Question nine asked students to estimate how many hours they sleep per day. There are three options: 1 (less than 7 h), 2 (7 h), and 3. (more than 7 h). In questions 10 to 14, students were asked how many times per week they did activities that made them sweat (e.g., stretching, muscle strengthening, walking, and cycling for at least 30 min). These activities were graded on a three-point scale of 1 (never), 2 (once), and 3 (more than two times).

#### 2.3.4 Toxic substance consumption

This section assesses respondents’ cigarette/waterpipe smoking habits and alcohol consumption. The items are scored on a three-point scale (i.e., No, Tried but stopped, and Yes).

### 2.4 Analysis

The statistical analyses were conducted using IBM SPSS Statistics for Windows, Version 25 (SPSS Inc., Chicago, IL, USA). Descriptive analyses were conducted for all study variables, including frequencies, percentages, means, and standard deviations. Independent sample *t*-test and the chi-square test were used to compare the groups. A p-value of <0.05 was set as the level of significance.

## 3 Results

### 3.1 Characteristics of study participants

A total of 766 students were invited to participate, 708 of whom completed the questionnaire and were included in the final analysis, and 58 were excluded due to incomplete responses. Of the 708 students, 647 (91.4%) were from public universities, and 61 (8.6%) were from private universities. About 443 of the total sample were female (62.6%), 179 (25.3%) were in their first year of university, 123 (17.4%) were in their second year, 219 (30.9%) were in their third year, 187 (26.4%) were in their fourth year or above. Most of the participants (90%, n = 637) were single. The mean age was 20.88(*SD* = 2.20), ranging from 18 to 37. About 506 (71.5%) were majoring in subjects unrelated to healthcare (i.e., science, computer science, engineering, humanities, law, business and economics, arts, or Islamic sciences). Only 202 (28.5%) were in healthcare majors (i.e., medicine, nursing, or pharmacy) (Table 1).

**Table 1:** Characteristics of study participants by student’s sex.

Characteristic	Category	*Males n* = 265 (37.4%)	*Females n* = 443 (62.6%)	Total *n* = 708 (100%)
University type	Public	231 (35.7.4%)	416 (64.3%)	647 (91.4%)
	Private	34(55.7%)	27 (44.3%)	61(8.6%)
Major	Health	49 (18.5%)	153(34.5%)	202 (28.5%)
	Non-Health	216 (81.5%)	290 (65.5%)	506 (71.5%)
Study level	1st Year	51 (19.2%)	128 (28.9%)	179 (25.3%)
	2nd Year	49 (18.5%)	74 (16.7%)	123 (17.4%)
	3rd Year	81 (30.6%)	137 (30.9)	219 (30.8%)
	4th Year and above	84 (31.7%)	103 (23.3%)	187 (26.4%)
Age, M(SD), range*		21.54 (2.58) 18-37	20.48(1.84), 18–35.	20.88(2.20), 18-37

Note: *M: mean, (SD): Standards Divination.

### 3.2 Food consumption in the last 24 h

Overall, 54.4% of the students were found to have consumed French fries or potato chips in the preceding 24 h, and 54% were found to have consumed cookies, doughnuts, pie, or cake at least once in the initial 24 h. About 52% of the students had not consumed vegetables, 48.2% had consumed green salad, and 47.3% had consumed fruit at least once in the preceding 24 h. Interestingly, 66.9% of the students indicated that they had not consumed hamburgers, hot dogs, or sausages in the initial 24 h (Table 2).

**Table 2:** Association of sex on food consumption among a sample of college students in Jordan.

Food consumption in the last 24 h	Male* *n* = 265	Female* *n* = 443	*P*^a^ value	Total^b^*n* = 708
**Fruits**				
No	103 (38.9%)	133 (30.0%)	0.044	236 (33.3%)
Yes, once	118 (44.5%)	217 (49.0%)		335 (47.3%)
Yes, twice or more	44 (16.6%)	93 (21.0%)		137 (19.4%)
**Fruit juice drinking**				
No	104 (39.2%)	185 (41.8%)	0.739	289 (40.8%)
Yes, once	122 (46.0%)	200 (45.1%)		322 (45.5%)
Yes, twice or more	39 (14.7%)	58 (13.1%)		97 (13.7%)
**Green Salad**				
No	108 (40.8%)	193 (43.6%)	0.756	301 (42.5%)
Yes, once	131 (49.4%)	210 (47.4%)		341 (48.2%)
Yes, twice or more	26 (9.8%)	40 (9.0%)		66 (9.3%)
**Cooked vegetables**				
No	138 (52.1%)	230 (51.9%)	0.165	368 (52.0%)
Yes, once	94 (35.5%)	176 (39.7%)		270 (38.1%)
Yes, twice or more	33 (12.5%)	37 (8.4%)		70 (9.9%)
**Hamburger, hot dogs, sausage**				
No	169 (63.8%)	305 (68.8%)	0.088	474 (66.9%)
Yes, once	73 (27.5%)	117 (26.4%)		190 (26.8%)
Yes, twice or more	23 (8.7%)	21 (4.7%)		44 (6.2%)
**French fries or potato chips**				
No	104 (39.2%)	146 (33.0%)	0.224	250 (35.3%)
Yes, once	134 (50.6%)	251 (56.7%)		385 (54.4%)
Yes, twice or more	27 (10.2%)	46 (10.4%)		73 (10.3%)
**Cookies, doughnuts, pie, or cake**				
No	96 (36.2%)	132 (29.8%)	0.149	228 (32.2%)
Yes, once	131 (49.4%)	251 (56.7%)		382 (54.0%)
Yes, twice or more	38 (14.3%)	60 (13.5%)		98 (13.8%)

*Note.* *Cell entries for male and female are percent for sex. ^a^*χ*^2^ test for independence. ^b^ Cell entries are frequencies and percentages for all respondents.

There was a significant association between students’ sex and fruit consumption. Female students consumed more fruits than male students (*p* = 0.044) (Table 2). However, there was no significant association in food consumption based on study major (i.e., health vs. non-health) (Table 3).

**Table 3:** Association of Study Major on food consumption among a sample of college students in Jordan.

Food consumed yesterday	Health-major* *n* = 202	Non- Health major* *n* = 506	*P*^a^ value
**Fruits**			
No	61 (30.2%)	175 (34.6%)	0.524
Yes, once	101 (50.0%)	234 (46.2%)	
Yes, twice or more	40 (19.8%)	97 (19.2%)	
**Fruit juice drinking**			
No	88 (43.6%)	201 (39.7%)	0.533
Yes, once	90 (44.5%)	232 (45.8%)	
Yes, twice or more	24 (11.9%)	73 (14.4%)	
**Green Salad**			
No	82 (40.6%)	219 (43.3%)	0.475
Yes, once	104 (51.5%)	237 (46.8%)	
Yes, twice or more	16 (7.9%)	50 (9.9%)	
**Cooked vegetables**			
No	103 (51.0%)	265 (52.4%)	0.264
Yes, once	84 (41.6%)	186 (36.8%)	
Yes, twice or more	15 (7.4%)	55 (10.9%)	
**Hamburger, hot dogs, sausage**			
No	146 (72.3%)	328 (64.8%)	0.105
Yes, once	48 (23.8%)	142 (28.1%)	
Yes, twice or more	8 (4.0%)	36 (7.1%)	
**French fries or potato chips**			
No	71 (35.1%)	179 (35.4%)	0.350
Yes, once	105 (52.0%)	280 (55.3%)	
Yes, twice or more	26 (12.9%)	47 (9.3%)	
**Cookies, doughnuts, pie, or cake**			
No	64 (31.7%)	164(32.4%)	0.885
Yes, once	108 (53.5%)	274 (54.2%)	
Yes, twice or more	30 (14.9%)	68 (13.4%)	

*Note.* *Cell entries for health and non-health major are percent for study major. ^a^*χ*^2^ test for independence.

### 3.3 Toxic substances consumption (cigarette, water pipe, and alcohol)

Table 4 displays the consumption of toxic substances among the total sample based on gender. Overall, 15.5% of the students were cigarette smokers, 16.7% were waterpipe smokers, and only 4.4% were alcohol consumers. The mean age at which the participants had started consuming toxic substances was 16.63(*SD* = 2.48) years for cigarette smoking, 17.64(*SD=*1.87) years for waterpipe smoking, and 18.03(*SD* = 1.49) for alcohol consumption.

**Table 4:** Association of sex on toxic substance consumption among a sample of college students in Jordan.

Toxic substance consumption	Male* *n* = 265	Female* *n* = 443	*P-*value	Total^c^*n* = 708
**Ever smoked Cigarettes**				
Yes, and still smoking	93 (35.1%)	17 (3.8%)	p < 0.001^a^	110 (15.5%)
Tried but stopped	58 (21.9%)	86 (19.4%)		144 (20.3%)
No	114 (43.0%)	340 (76.7%)		454 (64.1%)
Age of the first cigarette, Mean (SD)	16.45 (2.48)	17.59 (2.34)	0.083^**b**^	16.63 (2.48)
**Ever smoked waterpipe**				
Yes, and still smoking	72 (27.2%)	46 (10.4%)	p < 0.001^a^	118 (16.7%)
Tried but stopped	72 (27.2%)	121 (27.3%)		193 (27.3%)
No	121 (45.7%)	276 (62.3%)		397 (56.1%)
Age of first water pipe, Mean (SD)	17.28 (1.95)	18.20 (1.61)	0.009^b^	17.64 (1.87)
**Ever consumed Alcool**				
Yes, and still drinking	27 (10.2%)	4 (0.9%)	p < 0.001^a^	31 (4.4%)
Tried but stopped	27 (10.2%)	15 (3.4%)		42 (5.9%)
No	211 (79.6%)	424 (95.7%)		635 (89.7%)
Age of first Alcohol consumption, Mean (SD)	17.85 (1.48)	19.25 (0.95)	0.080 ^**b**^	18.03 (1.49)

*Note.* *Cell entries for males and females are percent for sex. ^a^*χ*^2^ test for independence. ^**b**^*t*-test of independence. ^C^ Cell entries are frequencies and percentages of all respondents.

The results revealed significant associations between male and female students consuming toxic substances. Male students were more likely than female students to be cigarette smokers (35.1% vs. 3.8%, *p* < 0.001), waterpipe smokers (27.2 vs.10.4%, *p* < 0.001), and alcohol consumers (10.2% vs. 0.9%, *p* < 0.001). Compared to females, males were also found to have started waterpipe smoking at an earlier age (17.28 years vs. 18.20 years, *p* = 0.009) (Table 4).

Furthermore, the results revealed that non-health major students consumed more cigarettes than health major students (18.6% vs. 7.9%, *p* < 0.001) and smoked waterpipes (18.8% vs. 11.4%, *p* = 0.027) (Table 5).

**Table 5:** Association of study major on toxic substances consumption among a sample of college students in Jordan.

Substance Consumption	Health-major* *n* = 202	Non- Health major * *n* = 506	*P*^*a*^ value
**Ever smoked Cigarettes**			
Yes, and still smoking	16 (7.9%)	94 (18.6%)	p < 0.001
Tried but stopped	37 (18.3%)	107 (21.1%)	
No	149 (73.8%)	305 (60.3%)	
Age of the first cigarette, Mean (SD)	16.69 (2.30)	16.62 (2.52)	0.917^b^
**Ever smoked waterpipe**			
Yes, and still smoking	23 (11.4%)	95 (18.8%)	0.027
Tried but stopped	65 (32.2%)	128 (25.3%)	
No	114 (56.4%)	283 (55.9%)	
Age of first water pipe, Mean (SD)	18.09 (1.99)	17.53 (1.83)	0.200^b^
**Ever consumed alcohol**			
Yes, and still drinking	4 (2.0%)	27 (5.3%)	0.104
Tried but stopped	10 (5.0%)	32 (6.3%)	
No	188 (93.1%)	447 (88.3%)	
Age of first Alcohol consumption, Mean (SD)	17.75 (.957)	18.07 (1.56)	0.693^b^

*Note.* *Cell entries for health and non-health are percent for major. ^a^*χ*^2^ test for independence. ^**b**^*t*-test of independence.

### 3.4 Physical activity and sedentary behaviors

Regarding physical activity, 58.2% of the students reported practicing sports, 15.0% doing sports regularly, 16.7% walking for more than 2 h per day, and 13.4% carrying out moderate activities for more than 2 h per day. Among the participating students, 9% reported spending more than 2 h watching television, 19.9% using the computer, 29.2% studying, and 19.1% commuting. Approximately 26.6% of the participants reported getting less than 7 h of sleep per night. As with regards to the participants’ sedentary behaviors in the preceding week, 52.8% of the students had not carried out any stretching activities, 62.4% had not carried out any strengthening activities, 35% had not done any cycling, and 68.4% had not taken part in any physical activity classes (Table 6).

**Table 6:** Association of sex on physical activity and sedentary behaviors among a sample of college students in Jordan.

Behavior	Male* *n* = 265	Female* *n* = 443	*P*^*a*^ value	Total^b^*n* = 708
**Perform any sport**				
Yes	194 (73.2%)	218 (49.2%)	p < 0.001	412 (58.2%)
No	71 (26.8%)	225 (50.8%)		296 (41.8%)
**Sports Frequency per week**				
Never	71 (26.8%)	225 (50.8%)	p < 0.001	296 (41.8%)
Twice a week or less	64 (24.2%)	70 (15.8%)		134 (18.9%)
Three to six times weekly	89 (33.6%)	83 (18.7%)		172 (24.3%)
Once daily	41 (15.5%)	65 (14.7%)		106 (15.0%)
**Television viewing per day**				
One hour or less	186 (70.2%)	282 (63.7%)	0.042	468 (66.1%)
Two hours or less	52 (19.6%)	124 (28.0%)		176 (24.9%)
More than 2 h	27 (10.2%)	37 (8.4%)		64 (9.0%)
**The computer stays per day**				
One hour or less	145 (54.7%)	269 (60.7%)	0.272	414 (58.5%)
Two hours or less	64 (24.2%)	89 (20.1%)		153 (21.6%)
More than 2 h	56 (21.1%)	85 (19.2%)		141 (19.9%)
**Studying frequency per day**				
One hour or less	106 (40.0%)	142 (32.1%)	0.045	248 (35.0%)
Two hours or less	94 (35.5%)	159 (35.9%)		253 (35.7%)
More than 2 h	65 (24.5%)	142 (32.1%)		207 (29.2%)
**Quiet activities per day**				
One hour or less	189 (71.3%)	319 (72.0%)	0.840	508 (71.8%)
Two hours or less	38 (14.3%)	67 (15.1%)		105 (14.8%)
More than 2 h	38 (14.3%)	57 (12.9%)		95 (13.4%)
**Walking per day**				
One hour or less	121 (45.7%)	254 (57.3%)	0.008	375 (53.0%)
Two hours or less	90 (34.0%)	125 (28.2%)		215 (30.4%)
More than 2 h	54 (20.4%)	64 (14.4%)		118 (16.7%)
**Time in transportation means**				
One hour or less	103 (38.9%)	212 (47.9%)	0.003	315 (44.5%)
Two hours or less	95 (35.8%)	163 (36.8%)		258 (36.4%)
More than 2 h	67 (25.3%)	68 (15.3%)		135 (19.1%)
**Number of sleeping hours**				
Less than 7 h	77 (29.1%)	111 (25.1%)	0.002	188 (26.6%)
Seven hours	115 (43.4%)	152 (34.3%)		267 (37.7%)
More than 7 h	73 (27.5%)	180 (40.6%)		253 (35.7%)
**Last week sweating during physical activity**				
Never	72 (27.2%)	176 (39.7%)	0.001	248 (35.0%)
Once to twice	95 (35.8%)	151 (34.1%)		246 (34.7%)
More than twice	98 (37.0%)	116 (26.2%)		214 (30.2%)
**Last week stretching activities**				
Never	105 (39.6%)	269 (60.7%)	p < 0.001	374 (52.8%)
Once to twice	94 (35.5%)	129 (29.1%)		223 (31.5%)
More than twice	66 (24.9%)	45 (10.2%)		111 (15.7%)
**Last week strengthening activities**				
Never	113 (42.6%)	329 (74.3%)	p < 0.001	442 (62.4%)
Once to twice	76 (28.7%)	67 (15.1%)		143 (20.2%)
More than twice	76 (28.7%)	47 (10.6%)		123 (17.4%)
**Last week rode a bicycle for 30 min**				
Never	78 (29.4%)	170 (38.4%)	0.051	248 (35.0%)
Once to twice	82 (30.9%)	124 (28.0%)		206 (29.1%)
More than twice	105 (39.6%)	149 (33.6%)		254 (35.9%)
**Last week physical activity class**				
Never	145 (54.7%)	339 (76.5%)	p < 0.001	484 (68.4%)
Once to twice	48 (18.1%)	52 (11.7%)		100 (14.1%)
More than twice	72 (27.2%)	52 (11.7%)		124 (17.5%)

*Note.* *cell entries for males and females are percent for sex. ^a^*χ*^2^ test for independence. ^b^ Cell entries are frequencies and percentages of all respondents.

In comparison to female students, male students were more likely to engage in sports activities (73.2% vs. 49.2%, *p* < 0.001), sweat due to physical activity (37.0% vs. 26.2%, *p* = 0.001), and carry out stretching exercises (24.9% vs. 10.2%, *p* < 0.001) in the preceding week. Male students were more likely than female students to spend more than 2 h in transportation (25.3% vs. 15.3%, *p* = 0.003) and walk for more than 2 h per day (20.4% vs. 14.4%, *p* = 0.008). However, female students were more likely than male students to spend 2 h or less per day watching television (28.0% vs. 19.6%, *p* = 0.042), to spend more than 2 h per day studying (32.1% vs. 24.5%, *p* = 0.045), and to sleep for more than 7 h per night (40.6% vs. 27.5%, *p* = 0.002) (Table 6).

Students in health majors were more likely than students in non-health majors to spend more than 2 h per day studying (44.6% vs. 23.1%, *p* < 0.001), to spend more than 2 h per day using the computer (25.7% vs. 17.6%, *p* = 0.007), and to sleep for more than 7 h per night (42.1% vs. 33.2%, *p* = 0.005) (Table 7). However, students in non-health majors were more likely than students in health majors to have carried out stretching activities (18.0% vs. 9.9%, *p* = 0 0.021). They participated in physical activity classes (20.0% vs. 11.4%, *p* = 0.02) more than twice in the preceding week (Table 7).

**Table 7:** Association of study major on physical activity and sedentary behaviors among a sample of college students in Jordan.

Behavior	Health major* *n* = 202	Non-Health major* *n* = 506	*P*^a^ value
**Perform any sport**			
Yes	115 (56.9%)	297 (58.7%)	0.667
No	87 (43.1%)	209 (41.3%)	
**Sports Frequency per week**			
Never	87 (43.1%)	209 (41.3%)	0.225
Twice a week or less	34 (16.8%)	100 (19.8%)	
Three to six times weekly	57 (28.2%)	115 (22.7%)	
Once daily	24 (11.9%)	82 (16.2%)	
**Television viewing per day**			
One hour or less	139 (68.8%)	329 (65.0%)	0.296
Two hours or less	50 (24.8%)	126 (24.9%)	
More than 2 h	13 (6.4%)	51 (10.1%)	
**Computer stay per day**			
One hour or less	100 (49.5%)	314 (62.1%)	0.007
Two hours or less	50 (24.8%)	103 (20.4%)	
More than 2 h	52 (25.7%)	89 (17.6%)	
**Studying frequency per day**			
One hour or less	48 (23.8%)	200 (39.5%)	p < 0.001
Two hours or less	64 (31.7%)	189 (37.4%)	
More than 2 h	90 (44.6%)	117 (23.1%)	
**Quiet activities per day**			
One hour or less	150 (74.3%)	358 (70.8%)	0.218
Two hours or less	32 (15.8%)	73 (14.4%)	
More than 2 h	20 (9.9%)	75 (14.8%)	
**Walking per day**			
One hour or less	115 (56.9%)	260 (51.4%)	0.256
Two hours or less	60 (29.7%)	155 (30.6%)	
More than 2 h	27 (13.4%)	91 (18.0%)	
**Time in transportation means**			
One hour or less	84 (41.6%)	231 (45.7%)	0.264
Two hours or less	83 (41.1%)	175 (34.6%)	
More than 2 h	35 (17.3%)	100 (19.8%)	
**Number of sleeping hours**			
Less than 7 h	37 (18.3%)	151 (29.8%)	0.005
Seven hours	80 (39.6%)	187 (37.0%)	
More than 7 h	85 (42.1%)	168 (33.2%)	
**Last week sweating during physical activity**			
Never	61 (30.2%)	187 (37.0%)	0.120
Once to twice	81 (40.1%)	165 (32.6%)	
More than twice	60 (29.7%)	154 (30.4%)	
**Last week stretching activities**			
Never	110 (54.5%)	264 (52.2%)	0.021
Once to twice	72 (35.6%)	151 (29.8%)	
More than twice	20 (9.9%)	91 (18.0%)	
**Last week strengthening activities**			
Never	138 (68.3%)	304 (60.1%)	0.115
Once to twice	33 (16.3%)	110 (21.7%)	
More than twice	31 (15.3%)	92 (18.2%)	
**Last week rode a bicycle for 30 min**			
Never	72 (35.6%)	176 (34.8%)	0.976
Once to twice	58 (28.7%)	148 (29.2%)	
More than twice	72 (35.6%)	182 (36.0%)	
**Last week physical activity class**			
Never	146 (72.3%)	338 (66.8%)	0.022
Once to twice	33 (16.3%)	67 (13.2%)	
More than twice	23 (11.4%)	101 (20.0%)	

Note. *Cell entries for health and non-health major are percent for study major. aχ2 test for independence.

## 4 Discussion

This study aimed to assess university students’ food consumption, physical activity, smoking, and alcohol use based on sex and major of study. Our findings showed that more than half of the participating students were found to have consumed French fries or potato chips, cookies, doughnuts, or pie or cake at least once in the preceding 24 h. This finding aligns with previous national surveys conducted among Jordanian students [38] and Lebanese students [37]. The results of this study can be explained by the availability and accessibility of fast food in and around the university environment. University students have a limited variety of nutrients because the food choices in the university environment are not usually the healthiest options, which increases the risk of weight gain and chronic diseases among students.

The present study also revealed that 48% of the students had consumed fruit and green salad at least once in the preceding 24 h. Although this may seem a high percentage for our sample, previous studies have suggested it reflects fruit and vegetable consumption frequency but not portions. Therefore, it is likely that the participating students consumed less fruit and vegetables than the nine daily servings recommended by the American College Health Association guidelines [39]. The result is consistent with a survey of 6727 American college students, which found only 8.5% reported eating five or more servings of fruit and vegetables daily [39].

Therefore, university students often consume fast food more frequently than fruit and vegetables. A study found that the most influencing fruit and vegetable intake during emerging adulthood is the favorable test preferences, availability of fruit and vegetables, and perceived time barriers to healthy eating [29]. University students often struggle with time management between the commitments of study responsibilities, work, engaging in other social activities, and the low availability of fruit and vegetables in the university environment where the students spend a lot of their time. It seems that university students do not consider having a healthy diet the main priority. They do not have the self-regularity skills required for maintaining a nutritious diet, such as controlling their shopping and preparing meals [40].

Moreover, the results of this study revealed no significant differences between students in food frequency by gender and major of study. Similar results were found by Salameh et al. [37] among students in Lebanon that the major of study is not a determinant factor in the food consumption of university students.

The transitional stage from adolescence to young adulthood can explain the insignificant differences between students by gender, in which all students share similar challenges in a new environment. This transition from school to the university creates an environment that negatively affects food consumption. The increase in academic requirements, stress, limited time management skills, the quality of the food served in the cafeteria, and changes in family support led to changes in university students’ food consumption.

Regarding consuming toxic substances (cigarettes, waterpipe, and alcohol), our results indicated that 16% of the students were cigarette smokers, 17% were waterpipe smokers, and 5% were alcohol consumers. These findings are similar to those reported by previous studies [10,21,40]. Compared to cigarette smoking and alcohol consumption, water pipe smoking was highest among the students. This may be attributed to waterpipe smoking being perceived as less harmful than cigarette smoking and is socially acceptable and expected behavior [41].

In this study, the low prevalence of alcohol consumption reflects the Islamic beliefs widely adopted in Jordan. Alcohol consumption is prohibited in Islam, stigmatized, and considered socially unacceptable in Arab culture. Therefore, alcohol consumers are condemned by society [10]. However, due to the topic’s sensitivity, the participants’ answers regarding alcohol consumption may have been impacted by self-reporting bias.

In the current study, the prevalence of the consumption of toxic substances was higher among male students than female students. This finding corresponds with the results of many previous studies [20,21,37,42]. A survey conducted in Germany found that male university students were more likely than females to take drugs and consume alcohol. However, no gender-based differences in the rates of regular cigarette smoking were reported [43]. The results of a qualitative study indicated that society considers female smoking inappropriate and views female smokers as disreputable and out of control [44].

On the other hand, male smoking positively reflects masculinity, strictness, relaxation, and authoritativeness. Traditional middle eastern cultures view male smoking as more acceptable than female smoking [45]. Also, male students are often permitted to stay out late, have friends over when their parents are not home, and have a room where they can spend unsupervised. Spending long durations of time unsupervised increases the likelihood of toxic substance consumption among males [46]. However, the prevalence of toxic substance consumption among female students may be underestimated due to reporting bias.

The current study found cigarette and waterpipe smoking prevalence rates higher among non-health majors than among health majors. Meanwhile, no significant association between alcohol consumption and the significance of the study was identified. Previous studies have indicated that students from healthcare faculties are more likely to adopt healthy behaviors, such as avoiding toxic substance consumption, than non-healthcare faculties [28,29]. This can be explained by promoting healthy behaviors in many of the courses taught in healthcare faculties [28]. A previous national study found the prevalence rates of smoking among students from faculties of law and religious studies to be the lowest compared to those among students from other faculties [20].

Moreover, this study showed that the mean age of starting cigarette smoking was 17 years. This finding is consistent with the study among Jordanian students [20]. Another study among Jordanian students reported that the age of starting cigarette and water pipe smoking ranged from 18 to 21 years. While the average age of starting cigarettes, waterpipe, and alcohol among Lebanese university students ranged from 14.4 to 16.4 years, which is younger than that of the current study. Most smokers start smoking before 18 years of age, and the initial exposure to smoking at a younger age is documented as a risk factor for heavier smokers as adults.

This study revealed that 58% of the participating undergraduate students practiced sports regularly, similar to the rate reported among students in Lebanon [37]. Despite the availability of numerous options for physical activities at university, students often do not have the time to regularly participate in physical activity programs. In our study, male students reported practicing sports more frequently than females. Previous studies support this finding [37,47]. This result may be explained by the fact that females face barriers that prevent them from exercising, such as difficulty exercising in the streets and poor access to sports centers [25]. Females also have lower self-efficacy and negative beliefs about their physical abilities [48].

### 4.1 Limitations

There were some limitations in this study. The use of self-reported instruments is subjected to social desirability responses, which may underestimate ’students’ responses to the alcohol and substance use questions. Although we have a representative sample of undergraduate students from five public and private universities, the findings' generalizability is limited to students in other Jordanian institutions. Further, approximately two-thirds of the participants (63%) were females, explaining the gender-based differences regarding health-risk behaviors. In this study, the main concern was to examine the main risk behaviors associated with increased risk of non-communicable diseases and harm students’ physical and mental health. However, some other risk behaviors are important to identify in future research. In our analysis, gender and study majors were used as covariates; other socio-demographic variables may be necessary for determining group differences.

## 5 Conclusion

Our findings revealed several patterns of health-risk behaviors among undergraduate students in Jordan. Specifically, most students were likely to engage in health risk behaviors, such as inadequate fruit and vegetable consumption, insufficient physical activity, smoking, water pipe, and alcohol use. There were significant differences between students based on their gender and study majors in how they engaged in health-related risk behaviors. Specifically, male students and students from non-health majors were more likely to engage in health-related risk behaviors. Our findings highlight the need for strengthening health services targeting health-promoting and protective behaviors and preventing health-risk behaviors among university students. More campus health promotion initiatives and programs are needed to target smoking, alcohol use, food consumption, and physical activity. The distinction between students in adopting health-related risk behaviors calls for tailored interventions considering students’ gender and their study majors. Integrating health-promoting behaviors and health-related risk behaviors into the curriculum for undergraduate students, particularly those majoring in health-related fields is important.

## Author contribution statement

Nahla Al Ali: Conceived and designed the experiments; Performed the experiments; Analyzed and interpreted the data; Contributed reagents, materials, analysis tools or data; Wrote the paper.

Fatima Khazaaleh: Conceived and designed the experiments; Performed the experiments; Contributed reagents, materials, analysis tools or data; Wrote the paper.

## Data availability statement

Data will be made available on request.

## Funding

This research was supported by internal funding from the 10.13039/501100019004Deanship of Research, Jordan University of Science and Technology [Research Grant No: 20160232].

**Consent to Participate**: Informed consent was obtained from all participants in the study.

**Ethics Approval:** The Institutional Review Board approved the Jordan University of Science and Technology (No 33/96/2016, dated May 24, 2016).

## Declaration of competing interest

The authors declare that they have no known competing financial interests or personal relationships that could have appeared to influence the work reported in this paper.
